# MGMR: leveraging RNA-Seq population data to optimize expression estimation

**DOI:** 10.1186/1471-2105-13-S6-S2

**Published:** 2012-04-19

**Authors:** Roye Rozov, Eran Halperin, Ron Shamir

**Affiliations:** 1 The Blavatnik School of Computer Science, Tel-Aviv University, Tel Aviv 69978, Israel; 2 Molecular Microbiology and Biotechnology Department, Tel-Aviv University, Tel Aviv 69978, Israel; 3 International Computer Science Institute, Berkeley, CA, 94704, USA

## Abstract

**Background:**

RNA-Seq is a technique that uses Next Generation Sequencing to identify transcripts and estimate transcription levels. When applying this technique for quantification, one must contend with reads that align to multiple positions in the genome (multireads). Previous efforts to resolve multireads have shown that RNA-Seq expression estimation can be improved using probabilistic allocation of reads to genes. These methods use a probabilistic generative model for data generation and resolve ambiguity using likelihood-based approaches. In many instances, RNA-seq experiments are performed in the context of a population. The generative models of current methods do not take into account such population information, and it is an open question whether this information can improve quantification of the individual samples

**Results:**

In order to explore the contribution of population level information in RNA-seq quantification, we apply a hierarchical probabilistic generative model, which assumes that expression levels of different individuals are sampled from a Dirichlet distribution with parameters specific to the population, and reads are sampled from the distribution of expression levels. We introduce an optimization procedure for the estimation of the model parameters, and use HapMap data and simulated data to demonstrate that the model yields a significant improvement in the accuracy of expression levels of paralogous genes.

**Conclusions:**

We provide a proof of principal of the benefit of drawing on population commonalities to estimate expression. The results of our experiments demonstrate this approach can be beneficial, primarily for estimation at the gene level.

## Introduction

With the rapid decline in the cost of sequencing, RNA-Seq has emerged as a legitimate competitor to mi-croarrays as a means of assessing global gene expression. Even as arrays currently enjoy a cost advantage, many new applications of information accessible only through sequencing further strengthen the case that sequencing may soon supplant arrays as the technology of choice for transcription analysis. One such application is fine-grained assessment of variation in expression and the sources for such variation, as exemplified by recent large-scale RNA-Seq studies [1,2] of two different HapMap [3] populations. Such studies complement genomic DNA sequencing by elucidating the link between SNPs and expression.

Unfortunately, with any new technology come its limitations, and several studies have pointed out that RNA-Seq is not immune to bias [4-6]. Perhaps the most widely discussed hurdle to accurate estimation in the case of RNA-Seq is the problem of reads mapped to multiple locations in the target genome (or in the target transcript sequences). These reads, which are called *multireads*, can stem from either paralogous gene sequences or from different isoforms of the same gene that share exons.

Several methods have emerged to address the multiread problem for paralog and isoform estimation [7-10]. These methods are all based on probabilistic modeling that is optimized by an expectation maximization procedure. It has been repeatedly shown that by using such methods one can get better quantification of the expression levels compared to quantification based on naive approaches of read assignment.

In many applications, a set of samples is studied. For instance, one may be interested in comparing the expression levels in cases verses controls, or in tissues originating from different organs. In such cases, it is plausible that the commonality of expression patterns within each of the defined groups of studied samples may be used to improve the quantification results in each of the samples. We demonstrate that by analyzing expression profiles of a population together, one gets expression estimates more accurate than those obtained by estimating individual sample expression levels independently. We describe and implement a probabilistic model of the sequencing process that incorporates population commonalities, and demonstrate its advantages over existing methods in the population setting.

## Methods

### RNA-Seq multiread expression estimation

As we seek to extend the prevalent generative model of RNA-Seq [7-11], we begin by reviewing the basic elements of that model. Let *G *= (*G*_1_, *...,G*_*M*_) be the set of M transcribed regions considered and *P *= (*P*_1_, *...,P*_*M*_) be the proportions of RNA bases attributed to each transcript out of the total number of transcribed bases in a sequenced sample. Regions may be either genes or transcripts, depending on the level of transcription being investigated. We require P to satisfy ∑_*gϵG *_*P*_*g *_= 1 and ∀*gϵG*, 0 ≤ *P*_*g *_≤ 1.

The model describes an RNA sequencing experiment where regions in G are randomly chosen according to the distribution P, start positions in these regions are chosen uniformly, and reads of length *ℓ *are generated by copying *ℓ *consecutive bases from each chosen region to produce a set of reads *R *= (*r*_1_,..., *r*_*ρ*_). Sequencing is assumed to be error prone, leading to a certain probability of error for each read base. Based on the repetitions present in the set of regions and errors in alignment, reads may fail to map to the region from which they originate or may map to additional locations. Thus, we assign a probability of obtaining read *r*_*j *_given that it originated from region $G_{k},P\left(r_{j}|G_{k}\right)\equiv \frac{{\left(1-\varepsilon \right)}^{\left(\ell -error_{jk}\right)}\varepsilon^{error_{jk}}}{\ell_{k}}$. In this case we rely on the alignment of *r*_*j *_to *G*_*k *_to afford us the best match position instead of summing over all possible starting positions. *ℓ*_*k *_is the effective length of *G*_*k *_(i.e., the number of start positions from which a full length read can be derived) as defined in [11], *ϵ *is taken to be a constant per-base error rate, errors are assumed to be independent, and *error*_*jk *_is the number of mismatches in the best alignment of *r*_*j *_to *G*_*k*_.

This formulation leads to the likelihood of observing the data:

(1) $$L\left(P;R\right)=\overset{\rho }{\underset{j=1}{\prod }}P\left(r_{j}|G,P\right)=\overset{\rho }{\underset{j=1}{\prod }}\overset{M}{\underset{k=1}{\sum }}P\left(G_{k}\right)P\left(r_{j}|G_{k}\right)$$

This likelihood function is used to estimate P given the read alignments. Typically, one will use expectation maximization to find the P for which the likelihood is maximized. It is assumed that *P*(*r*_*j*_|*G*_*k*_) is zero for all regions to which *r*_*j *_is not aligned.

### Common population extension

To estimate expression levels in N individuals from a defined population, we modify the above model by assuming that samples are drawn from a common population. This is imposed by having *P *= [(*P*_11_, ...,*P*_1*M*_), .., (*P*_*N*1 _*...*,*P*_*N M*_)] be probability densities drawn from a common Dirichlet distribution, defined by a set of hyper-parameters specific to the population: ∀*iϵ*[1, *N*], **p**_**i **_= (*P*_*i*1_,..., *P*_*iM*_) ~ *Dirichlet *(*α*_1_,..., *α*_*M*_).

For sample i, we denote the set of reads as $R_{i}=\left(r_{i1},\ldots ,r_{i\rho_{i}}\right)$, where each *r*_*ij *_is mapped to one or more regions in G. The output of a read alignment program defines the set of accepted regions for the read (in practice only alignments with up to 2 errors are accepted) and provides the number of errors in alignment for each read-region pair. This allows us to calculate *P*(*r*_*ij*_|*G*_*k*_) as done above for one sample. For convenience we denote *P*(*r*_*ij*_*|G*_*k*_) = *q*_*ijk *_(taken to be zero for all regions not mapped to), which is independent of ***α ***and ***P***.

As before, our objective is to estimate P, but in this case we must optimize by estimating P and ***α ***together. We begin by writing the likelihood function:

(2) $$L\left(p_{1},\ldots ,p_{N},\alpha ;R\right)=Pr\left(p_{1},\ldots ,p_{N}|\alpha \right)Pr\left(R|p_{1},\ldots ,{\text{p}}_{N}\right)$$

Since expression values are sampled from the Dirichlet distribution,

(3) $$Pr\left(p_{1},\ldots ,p_{N}|\alpha \right)=\overset{N}{\underset{i=1}{\prod }}P\left(p_{i}|\alpha \right)=\overset{N}{\underset{i=1}{\prod }}C\left(\alpha \right)\overset{M}{\underset{k=1}{\prod }}P_{ik}^{\alpha_{k}-1}$$

Where

(4) $$C\left(\alpha \right)\equiv \frac{\Gamma \left(\underset{k}{\sum }\alpha_{k}\right)}{\underset{k}{\prod }\Gamma \left(\alpha_{k}\right)}$$

and similar to (1) above,

(5) $$Pr\left(R|p_{1},\ldots ,p_{N}\right)=\overset{N}{\underset{i=1}{\prod }}\overset{\rho_{i}}{\underset{j=1}{\prod }}\overset{M}{\underset{k=1}{\sum }}P_{ik}q_{ijk}$$

This leads to

(6) $$L\left(p_{1},\ldots ,p_{N},\alpha ;R\right)=\left[\overset{N}{\underset{i=1}{\prod }}C\left(\alpha \right)\overset{M}{\underset{k=1}{\prod }}P_{ik}^{\alpha_{k}-1}\right]\left[\overset{N}{\underset{i=1}{\prod }}\overset{\rho_{i}}{\underset{j=1}{\prod }}\overset{M}{\underset{k=1}{\sum }}P_{ik}q_{ijk}\right]$$

Taking the log, we get

(7) $$\begin{array}{ll} log\left[L\left(p_{1},\ldots ,p_{N},\alpha ;R\right)\right] & =NlogC\left(\alpha \right)+\overset{M}{\underset{k=1}{\sum }}\left(\alpha_{k}-1\right)\overset{N}{\underset{i=1}{\sum }}logP_{ik}\;\\ & +\overset{N}{\underset{i=1}{\sum }}\overset{p_{i}}{\underset{j=1}{\sum }}\left(log\left(\overset{M}{\underset{k=1}{\sum }}P_{ik}q_{ijk}\right)\right)\;\\ & \; \end{array}$$

### Multi-Genome Multi-Read (MGMR) algorithm

We wish to estimate ***α ***and **p**_**1**_,...,**p**_**n **_to maximize equation (7) above. For this purpose, we adopt an alternating iterative procedure of estimating ***α ***given the current estimate of **p**_**1**_,...,**p**_**n **_and vice-versa until the total change in ***α ***becomes sufficiently small (or until a pre-set number of iterations have been executed).

Although for EM-based estimation methods convexity guarantees an optimal solution will be obtained, here (as shall be seen below) we have no such guarantee. Thus, we confine updates to be local by performing only one update for P and one for ***α***. By one MGMR iteration, we refer to one EM-based P update followed by one ***α ***update.

#### Estimating P given α

If we assume ***α ***is given, we can write the EM steps to find **p**_**1**_,...,**p**_**N**_:

**E step **Letting ***Match ***signify a matching between reads and regions, and *Match(j) *be the region from which read j originates, we get:

(8) $$\begin{array}{ll} log\left[L\left(P,\alpha ;R,\mathrm{Match}\right)\right] & =NlogC\left(\alpha \right)+\overset{M}{\underset{k=1}{\sum }}\left(\alpha_{k}-1\right)\overset{N}{\underset{i=1}{\sum }}logP_{ik}\;\\ & +\overset{N}{\underset{i=1}{\sum }}\overset{\rho_{i}}{\underset{j=1}{\sum }}\left(log\left(P_{iMatch\left(j\right)}{q_{ij}}_{Match\left(j\right)}\right)\right)\;\\ & \; \end{array}$$

which leads to

(9) $$Q\left(P,\alpha |P^{\left(t\right)},\alpha^{\left(t\right)}\right)=E_{M\;atch|R,P^{\left(t\right)},\alpha^{\left(t\right)}}\left[log\left(L\right)\right]$$

(10) $$\begin{array}{ll} =NlogC\left(\alpha^{\left(t\right)}\right) & +\overset{M}{\underset{k=1}{\sum }}\left(\alpha_{k}^{\left(t\right)}-1\right)\overset{N}{\underset{i=1}{\sum }}logP_{ik}\;\\ & +\overset{N}{\underset{i=1}{\sum }}\overset{\rho_{i}}{\underset{j=1}{\sum }}\overset{M}{\underset{k=1}{\sum }}\left(logP_{ik}+logq_{ijk}\right)*\frac{P_{ik}^{\left(t\right)}q_{ijk}}{\sum_{k=1}^{M}P_{ik}^{\left(t\right)}q_{ijk}}\;\\ & \; \end{array}$$

(11) $$\begin{array}{ll} =NlogC\left(\alpha^{\left(t\right)}\right)+\overset{M}{\underset{k=1}{\sum }}\left(\alpha_{k}^{\left(t\right)}-1\right)\overset{N}{\underset{i=1}{\sum }}logP_{ik} & +\overset{N}{\underset{i=1}{\sum }}\overset{\rho_{i}}{\underset{j=1}{\sum }}\overset{M}{\underset{k=1}{\sum }}a_{ijk}logP_{ik}\;\\ & +\overset{N}{\underset{i=1}{\sum }}\overset{\rho_{i}}{\underset{j=1}{\sum }}\overset{M}{\underset{k=1}{\sum }}a_{ijk}logq_{ijk}\;\\ & \; \end{array}$$

where

(12) $$a_{ijk}\equiv \frac{P_{ik}^{\left(t\right)}q_{ijk}}{\sum_{k=1}^{M}P_{ik}^{\left(t\right)}q_{ijk}}$$

**M step **Given that each *q*_*ijk *_is fixed, the above reduces to maximizing

(13) $$\begin{array}{ll} NlogC\left(\alpha^{\left(t\right)}\right) & +\overset{M}{\underset{k=1}{\sum }}\left(\alpha_{k}^{\left(t\right)}-1\right)\overset{N}{\underset{i=1}{\sum }}logP_{ik}\;\\ & +\overset{N}{\underset{i=1}{\sum }}\overset{\rho_{i}}{\underset{j=1}{\sum }}\overset{M}{\underset{k=1}{\sum }}a_{ijk}*logP_{ik}\;\\ & \; \end{array}$$

Using Lagrange multipliers and differentiating, we see that this is maximized with

(14) $$P_{ik}^{\left(t+1\right)}=\frac{\alpha_{k}^{\left(t\right)}-1+\sum_{j}a_{ijk}}{\sum_{k}\left(\alpha_{k}^{\left(t\right)}-1+\sum_{j}a_{ijk}\right)}$$

#### Estimating α given P

Given a new estimate for *P*^(*t*)^, we can use a fixed point iteration [12] to get a new estimate of ***α***

(15) $$\begin{array}{ll} F\left(\alpha \right) & =N\left[log\Gamma \left(\underset{k}{\sum }\alpha_{k}\right)-\underset{k}{\sum }log\Gamma \left(\alpha_{k}\right)\right]\;\\ & +\overset{M}{\underset{k=1}{\sum }}\left(\alpha_{k}-1\right)\overset{N}{\underset{i=1}{\sum }}logP_{ik}^{\left(t\right)}+Const\left(P^{\left(t\right)}\right)\;\\ & \; \end{array}$$

By using the known bound $\Gamma \left(x\right)\geq \Gamma \left(\hat{x}\right)exp\left(\left(x-\hat{x}\right)\Psi \left(x\right)\right)\;\left(\text{having}\Psi \left(x\right)=\frac{dlog\Gamma \left(x\right)}{dx}\right)$, we can get a lower bound on F(*α*):

(16) $$\begin{array}{l} F(\alpha )\geq N[(\sum_{k}\alpha_{k})\Psi (\sum_{k}\alpha_{k}^{\left(t\right)})-\sum_{k=1}^{M}log\Gamma (\alpha_{k})\\ \;\;\;\;\;\;\;\;\;\;\;\;+\sum_{k=1}^{M}\alpha_{k}log{\bar{P}}_{k}^{\left(t\right)}+Const(P^{\left(t\right)}) \end{array}$$

where $log{\bar{P}}_{k}=\frac{1}{N}*\sum_{i}logP_{ik}$.

We maximize this bound with a fixed point iteration similar to EM, noting that for fixed values of *P *convergence is guaranteed, and that for the Dirichlet distribution the maximum is the only stationary point [12]. This leads to the update

(17) $$\alpha_{k}^{\left(t+\right)}=\Psi^{-1}\left[\Psi \left(\sum \overset{\left(t\right)}{\underset{k}{\alpha }}\right)+log\overset{\left(t\right)}{\underset{k}{\bar{P}}}\right]$$

#### Heuristics/Implementation

**Figure 1:**
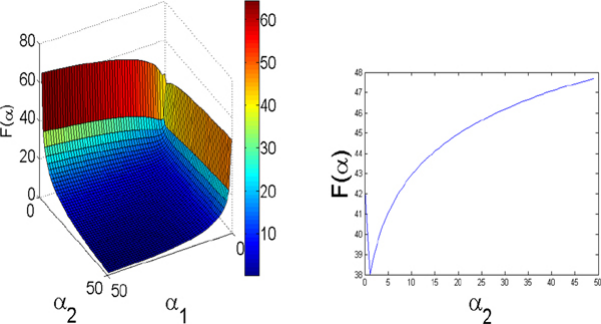
**A mesh representation of F(α) [equation (15)] showing non-convex behavior**. *P *is a 10 × 2 constant matrix and *α *is varied on [0:50,0:50]. The case shown is for N = 10, M = 2 (ten samples, two *α *parameters). Non-convex behavior is demonstrated by the values on the plane defined by α_1 _= .06 on the range [0,50] on the right.

As we have found *F*(**α**) presented in equation (15) is non-convex even in 2 dimensions (Figure 1), we confine updates to be local by allowing only one update for both the *α *and *P *estimation steps at each MGMR iteration. For genes with EM expression estimates equaling zero in all samples we substitute 10^-20 ^for their values in MGMR to avoid taking the log of zero. For P updates (e.g., equation 14), we avoid potentially negative P values by adding one to each alpha (thus ignoring -1 in the numerator and denominator). We implemented the algorithm in MATLAB, where the inputs required are read-gene map files for each sample as in SEQEM [7], and an initial P estimate matrix. Alphas are initialized as an M-length vector of ones.

## Results

### Experimental setup

As in [7,9,10], we examined MGMR's accuracy by comparing its estimates of known expression levels with those of existing methods, namely SEQEM [7] and RSEM [9,10]. The initial "known" expression levels were estimates obtained from RNA-Seq samples; how these estimates were obtained is described below. In our case, we had to simulate a population sharing similar expression levels and the same set of gene regions. Our experiment differed in that we sought to use additional information to improve on the estimates of these existing methods. These methods were designed to estimate expression of single samples, and each had specific advantages which we disregarded in our comparison. For example, we ignored both SEQEM's ability to utilize SNP information and RSEM's ability to allow estimation on assembled transcripts by using only reference sequences.

### Simulating data

To derive artificial reads, we first estimated expression on real biological samples using one method and then used the resulting distribution of expression values to generate simulated datasets for testing. Real samples were drawn from lymphoblastoid cells of the Yoruba in Ibadan (YRI) population [2,3]. As MGMR requires initial expression estimates, the estimate derived from the method it was being compared with in each case was input to MGMR. Thus, the four initial estimates used were from SEQEM, MGMR(SEQEM) (namely, MGMR initialized by SEQEM's result), RSEM, and MGMR(RSEM) (namely, MGMR initialized by RSEM estimates). We denote these four estimates SEQEM-A, SEQEM-B, MGMR-A and MGMR-B, respectively. We simulated reads by randomly selecting start sites falling within gene boundaries and extracting sequences from those positions. Read lengths were defined for each experiment, and simulations were repeated multiple times to account for randomness in the sampling process.

To derive the sequence set for the SEQEM comparison, we expanded upon the procedure used in [7]. There, SEQEM was shown to improve estimation of paralogous gene expression on a set of exon sequences from 51 Homo Sapiens chromosome 1 paralogs from the HomoloGene [13] database. We extracted a larger set of sequences containing all HomoloGene paralogs in Homo Sapiens having at least one exon longer than twice the read length used that do not overlap in genomic coordinates. We required this minimal length because sequences were sampled from exons, and we needed to ensure enough positions existed for full length reads to be sampled from these exons. 285 such genes remained (for reads of length 35 bp), and these were the genes on which expression was tested and from which read sequences were derived. The SEQEM-A and SEQEM-B read sets were generated based on randomly selected exons from these genes and the expression levels from the SEQEM-A and SEQEM-B estimates taken on 20 YRI individuals. The read length of 35 bp corresponded to that of the YRI samples, and a coverage level of 20 was chosen, as this was the level at which SEQEM was shown to perform best in [7]. We performed a total of 30 repetitions of read simulations, where each repetition consisted of 20 samples (corresponding to the original 20 YRI samples used).

For the RSEM-A and RSEM-B read sets, the transcript set used was also obtained by filtering the HomoloGene database to avoid gene overlaps, but no length filtering was required: reads were now sampled directly from transcripts which all had effective lengths greater than the read length used. 524 transcripts corresponding to 265 genes survived this filtering. For these read sets, we produced 30 repetitions of 74 samples, where each consisted of 100 bp reads at a coverage level of 20. In all other respects the sampling process and read generation steps were identical to those performed for the SEQEM-A and SEQEM-B read sets.

### Error measures

Accuracy was assessed by three error measures, the first two of which were applied in [7]: error rate, computed as $\frac{1}{n}\sum_{i}\frac{\left|P_{i}-Q_{i}\right|}{Q_{i}},\chi^{2}$ difference, computed as $\sum_{i}\frac{{\left(P_{i}-Q_{i}\right)}^{2}}{Q_{i}}$, and Kullback-Leibler divergence, computed as $\sum_{i}P_{i}\text{log}\frac{P_{i}}{Q_{i}}$. Here P is the estimated distribution generated by the tested algorithm and Q is the true distribution. Error measures were averaged over all repetitions per sample, and then over all samples.

### Simulated data with priors based on real estimates - estimating paralogous gene expression

To test performance on paralogous gene estimates, we set out to compare independent sample SEQEM estimates with MGMR's common population estimates. Before applying SEQEM, we looked to address one criticism of it from [11], where it was suggested that SEQEM's estimation could be improved by incorporation of transcript length correction. Upon examination, we found the effect of this correction was an increase in accuracy, and thus we maintained it for subsequent tests. This improvement is documented in the appendix.

**Figure 2:**
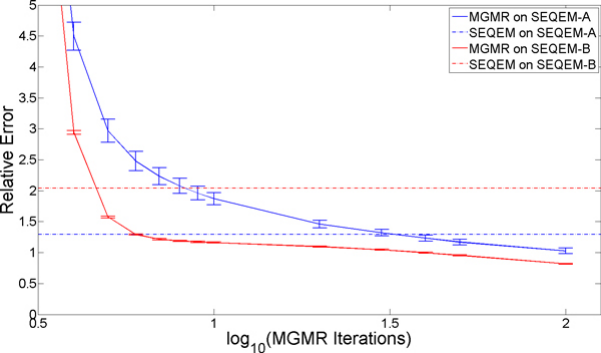
**Relative error measured on SEQEM-A and SEQEM-B data sets**. MGMR outputs on SEQEM-A and SEQEM-B initializations were compared with SEQEM up to 100 iterations. MGMR outputs were recorded at 1-10, 20, 30, 40, 50 and 100 iterations. The first few iterations have been trimmed to allow a compact presentation.

With this correction in place, we estimated expression levels on the SEQEM-A and SEQEM-B read sets, applying SEQEM and MGMR to each. Outputs were recorded at 1-10, 20, 30, 40, 50 and 100 iterations for MGMR and at 100 iterations for SEQEM. The results are shown in Figure 2. We observed that both error and variance levels dropped sharply within just a few iterations for MGMR, and converged to significantly better estimates on average than SEQEM. These trends were consistent across all three error measures [Table 1]. Variance seemed to diminish more with MGMR over time, as might be expected for a method that shares information across samples. Notably, MGMR outperformed SEQEM on estimates for samples based on SEQEM-A, where sample estimates were obtained independently (and thus we expect the variation inherent in the real samples to be maintained).

**Table 1:** MGMR vs. SEQEM error at 100 iterations on SEQEM-A and SEQEM-B data sets

	SEQEM-A sampling				SEQEM-B sampling			
	
	SEQEM		MGMR		SEQEM		MGMR	
	
	Error	SD	Error	SD	Error	SD	Error	SD
E	1.27	1 * 10^-2^	1.03	0.14	1.50	0.70	0.82	6 * 10^-3^
χ^2^	0.66	2 * 10^-3^	0.22	4 * 10^-3^	0.69	0.05	0.27	1 * 10^-4^
KL	0.29	7 * 10^-4^	0.14	1 * 10^-4^	0.18	2 * 10^-4^	0.17	1 * 10^-4^

These data sets were derived from SEQEM and MGMR(SEQEM) estimates, respectively, on 20 YRI samples. (E: relative error rate; χ^2^: Chi-squared error; KL: Kullback-Liebler divergence; SD: standard deviation)

### Simulated data with priors based on real samples - estimating transcript level expression

We also sought to examine whether MGMR can improve results in the more challenging setting of estimating transcript level expression. Here, we expect estimates to be noisier due to low expression values in the real samples, and we must contend with multiread mappings due to paralogous genes as well as to isoforms of particular genes sharing subsequences as a result of alternative splicing. In anticipation of this challenge, we used a larger set consisting of 74 sample of single-end YRI samples as the real data source and simulated 100 bp reads instead of 35 bp. This was expected to be a difficult case for estimation, as all genes in the set are paralogs and many have multiple isoforms, as described in the section "Simulating data."

Once expression estimation was performed on the YRI samples and read sets RSEM-A and RSEM-B were generated, we again performed expression estimates with RSEM and MGMR on each set. In this case, unfortunately, we found the results did not exhibit a consistent trend as before and overall appeared inconclusive. These results are summarized in Table 2. It remains to be seen why the error results differ according to the level of estimation (gene vs. transcript) performed.

**Table 2:** MGMR vs. RSEM error at 100 iterations on RSEM-A and RSEM-B data sets

	RSEM-A Sampling				RSEM-B Sampling			
	
	RSEM		MGMR		RSEM		MGMR	
	
	Error	SD	Error	SD	Error	SD	Error	SD
E	0.1	1 * 10^-3^	0.69	1 * 10^-3^	1.0	1 * 10^-4^	0.61	1 * 10^-3^
χ^2^	0.02	6 * 10^-4^	1.25	0.01	0.02	9 * 10^-4^	0.58	3 * 10^-4^
KL	1.5	0.22	0.6	1 * 10^-3^	0.8	0.11	0.38	6 * 10^-4^

E: relative error rate; χ^2^: Chi-squared error; KL: Kullback-Liebler divergence; SD: standard deviation

## Conclusion

As shown by the 1000 Genomes and HapMap projects, one of the drives of modern genetics and bioinformatics research is to characterize variation in populations. Because of cost and time constraints, such projects have only recently become feasible. In addition to such studies assessing genomic variation and its relation to disease phenotypes based on DNA, it is anticipated that RNA-Seq population studies will also grow in popularity to more directly assign functional significance to variant loci by means of transcription measures. Thus, it becomes essential to accurately measure the expression levels from each individual to characterize such variation. Here, we have shown that for one common study design an unexpected benefit can arise. When individuals in these studies are drawn from the same population, the estimates made on each can be made more accurate because of the commonalities among population members.

A shortcoming of the MGMR approach is that since it assumes commonality among the samples, outlier samples will be attracted towards the common denominator, and thus appear more similar to the group profile than they really are. In particular, if the data are subject to differential expression analysis, MGMR may reduce the number of differentially expressed genes.

We have investigated the efficacy of MGMR in tackling two typical experimental settings - inferring expression levels of paralogs at the gene level, and of isoforms (also drawn from a difficult set of paralogs). Although substantial gains were obtained in the first, more inquiry is required to demonstrate a benefit in the latter. It is worth noting that in each case at least a quarter of the regions considered showed improvement, as shown in Table 3. With these results, we submit a proof of concept that population structure can aid in estimation of expression levels for RNA-Seq samples.

**Table 3:** Proportion of genes for which MGMR improves estimates on different data sets

	SEQEM-A	SEQEM-B	RSEM-A	RSEM-B
Proportion	104/285	78/285	126/524	173/524
%	36.5	27.3	24.0	33.0

Proportions of regions (genes for SEQEM and transcripts for RSEM, respectively) for which MGMR has lower relative error on average than each method compared to.

## List of abbreviations

E: relative error rate; *χ*^2^: Chi-squared error; KL: Kullback-Liebler divergence; SD: standard deviation; bp: base pair

## Competing interests

The authors declare that they have no competing interests.

## Authors' contributions

RR and EH developed the method. RS and EH designed the experiments. RR implemented the method and performed experiments. RR, EH, and RS analyzed results and wrote the manuscript. All authors read and approved the final manuscript.
